# Effects of Temoporfin-Based Photodynamic Therapy on the In Vitro Antibacterial Activity and Biocompatibility of Gelatin-Hyaluronic Acid Cross-Linked Hydrogel Membranes

**DOI:** 10.3390/pharmaceutics14112314

**Published:** 2022-10-27

**Authors:** Kai-Chi Chang, Kuo-Chou Chiu, Wen-Cheng Chen, Wan-Chen Lan, Chi-Yuan Chen, Shih-Min Hsia, Tong-Hong Wang, Hsi-Feng Tu, Yin-Hwa Shih, Tzong-Ming Shieh

**Affiliations:** 1 School of Dentistry, China Medical University, Taichung 40402, Taiwan; 2 Division of Oral Diagnosis and Family Dentistry, Tri-Service General Hospital, National Defense Medical Center, Taipei 11490, Taiwan; 3 Advanced Medical Devices and Composites Laboratory, Department of Fiber and Composite Materials, Feng Chia University, Taichung 40724, Taiwan; 4 Department of Fragrance and Cosmetic Science, College of Pharmacy, Kaohsiung Medical University, Kaohsiung 80756, Taiwan; 5 Department of Healthcare Administration, Asia University, Taichung 41354, Taiwan; 6 Graduate Institute of Health Industry Technology, Chang Gung University of Science and Technology, Kweishan, Taoyuan 33303, Taiwan; 7 School of Nutrition and Health Sciences, Taipei Medical University, Taipei 110301, Taiwan; 8 Tissue Bank, Chang Gung Memorial Hospital, Taoyuan 33305, Taiwan; 9 Department of Dentistry, National Yang Ming Chiao Tung University, Taipei 112, Taiwan

**Keywords:** guided tissue regeneration, hydrogel membrane, photodynamic therapy, antibacterial, biocompatibility

## Abstract

This study was performed to design a hydrogel membrane that exhibits antibacterial properties and guides different tissues. Gelatin and hyaluronic acid were used as the main structures, 1-(3-dimethylaminopropyl)-3-ethylcarbodiimide hydrochloride (EDC) was used as a cross-linker, and temoporfin was used as an antibacterial agent. The results revealed that the hydrogel membrane impregnated with temoporfin (HM-T) had a fixation index of >89%. Temoporfin was used in conjunction with a diode laser and did not significantly affect EDC-induced cross-linking. The inhibitory activity of temoporfin showed that HM-T15 and HM-T30 (light exposure for 15 and 30 min, respectively) had remarkable antibacterial properties. The cell survival rate of HM-T15 was 73% of that of the control group, indicating that temoporfin exposure for 15 min did not exert cytotoxic effects on L-929 cells. HM and HM-T15 hydrogel membranes showed good cell adhesion and proliferation after 14 days of dark incubation. However, the hydrogel membrane containing temoporfin significantly reduced pro-inflammatory gene expression. In summary, the HM-T15 group showed potential as a biodegradable material for biocompatible tissue-guarded regeneration membranes with antibacterial properties. This study demonstrated the potential of temoporfin for innovative biomaterials and delivery systems applied to new regenerative periodontal therapies.

## 1. Introduction

Periodontitis is a common disease that leads to tooth loss [1]. Bacterial enzymes, endotoxins, and other cytotoxic factors may result in tissue destruction and cause chronic inflammation; chronic inflammation caused by bacterial infection is a major cause of periodontium destruction [2]. The clinical treatment of periodontitis involves removing plaques and controlling local inflammation [3,4]. However, removing all periodontal pathogens completely is difficult with conventional mechanical therapy because of the anatomical complexity of the roots of the teeth, especially in deep periodontal pockets, and bacterial invasion of the surrounding soft tissues [5,6,7,8]. PDT is widely used in medicine for a number of processes, such as marking lesions, shrinking tumors, and inhibiting microbial growth. With the limitations of traditional periodontal therapy, numerous attempts have been made to introduce antimicrobial photodynamic therapy (aPDT) as a common alternative to adjuvant treatment for chronic periodontitis [9,10,11].

aPDT is a treatment option that combines photophysical and photochemical processes in order to produce biological effects. When a photosensitizer (PS) binds to cells, it can be excited through illumination with a specific light wavelength, thereby producing cytotoxic reactive oxygen species (ROS) in the presence of ambient molecular oxygen. The oxidative stress that is caused by ROS and produced during illumination may lethally affect cancer cells and microbial pathogens [12,13]. An example of a highly effective second-generation synthetic PS is temoporfin, which elicits the same response to photodynamic therapy at lower levels and light doses than its first-generation predecessors. Temoporfin is one of the most potent clinically approved photosensitizers, whose hydrophobic character limits its aPDT effect. How to effectively deliver photosensitizers at the target site is an important issue. According to different application methods, optimized photodynamic therapy is continuously being developed. For example, aPDT has been combined with bacteriophages, biopolymers, drug and light delivery systems, efflux pump inhibitors, negative pressure systems, peptides, and sonodynamic therapy in dentistry [14]. The application of chemical reactions and oxygen-economical nano-photosensitizers are other strategies that have been utilized in order to improve aPDT outcomes [15,16]. aPDT treatment has been used for endodontic and periodontitis therapy in dentistry. Strategies to optimize aPDT treatment may vary due to the different sites of endodontic disease and periodontitis. In recent years, many studies have focused on the development of temoporfin-PDT, which requires nanoscale delivery systems, such as lipid- and polymer-based nanoparticles, carbon nanotubes, host–guest inclusion complexes, and conjugates [17,18]. 

Although these aPDT treatments can reduce symptoms and prevent disease progression, they cannot reconstruct the attachment of periodontal tissues to the original dental root and other periodontal tissues. Periodontal regeneration membranes combined with photodynamic therapy may provide more options to enhance antibacterial effects and promote tissue regeneration. Guided tissue regeneration (GTR) has been widely applied to periodontium regeneration in clinics; it is a regenerative surgical technique performed to cover adjacent soft tissues and to integrate bone [19,20]. Because bone and periodontal tissue formation is slower than soft tissue growth, GTR membranes can be used to keep a recovery space for regenerated tissues, prevent the inward overgrowth of gingival tissues, and provide spaces for periodontal ligaments and alveolar bone regeneration [21,22]. The resorbable GTR membrane degrades and adsorbs in the body gradually, so the patients do not need secondary surgery in order to remove the GTR membrane [23]. 

Gelatin exhibits high biocompatibility, biodegradability, cost-effectiveness, and low immunogenicity [24]. It has an arginylglycylaspartic acid sequence that is suitable for cell attachment and proliferation [25,26]. Thus, gelatin has been extensively used to repair periodontal tissues in dentistry. Similarly, hyaluronic acid has low immunogenicity, good biocompatibility, and unique viscoelasticity. It has been extensively utilized for ophthalmic vitreous bodies, artificial joint synovial fluid, and wound surface regeneration in order to treat osteoarthritis [27,28]. 

In this study, hyaluronic acid and gelatin were adopted as the main components of hydrogels, and 1-(3-dimethylaminopropyl)-3-ethylcarbodiimide hydrochloride (EDC) was used as a cross-linker to stabilize the mechanical properties of hydrogels based on hyaluronic acid and gelatin. aPDT was used as an antimicrobial protocol to decrease the risk of infection during periodontal tissue healing. The physical properties, antibacterial effects, and anti-inflammatory effects of the hydrogel membrane impregnated with temoporfin (HM-T) were analyzed.

## 2. Materials and Methods

### 2.1. Raw Materials

The following raw materials were used: gelatin (type B from bovine skin with an average molar mass of 40,000–50,000 g/mol; Sigma-Aldrich^®^, St. Louis, MO, USA), hyaluronic acid (molecular weight of 1800–2200 kDa; Kikkoman^®^, Tokyo, Japan), 1-(3-dimethylaminopropyl)-3-ethylcarbodiimide hydrochloride (EDC, molecular weight of 191.70 g/mole; Sigma-Aldrich^®^, St. Louis, MO, USA), and temoporfin (ChemScene, Monmouth Junction, NJ, USA). For the stock solution, 1 mg/mL of temoporfin solution was dissolved in dimethyl sulfoxide (DMSO) and was stored in the dark at −20 °C. When 1 mg/mL of temoporfin stock solution was diluted using PBS to 1 µg/mL temoporfin for hydrogel membrane soaking, there was almost no visible sediment.

### 2.2. Hydrogel Specimen Preparation

In accordance with previous studies and our preliminary research [29], gelatin (7 g) and hyaluronic acid (0.07 g) were mixed with 70 mL of 50 vol.% alcohol solution, and the mixture was heated to 55–60 °C with stirring for 1 h. The sample was modeled for 6 h, dehumidified at 25 °C for 24 h, soaked in a large quantity of 1% EDC cross-linker solution, kept in a refrigerator at 4 °C for 24 h of cross-linking, and rinsed with deionized water thrice for 30 min. Afterwards, EDC-cross-linked hydrogel membranes (HMs) were obtained. Phosphate-buffered saline (PBS) was used as a solvent to dissolve temoporfin. Each EDC-cross-linked hydrogel sample was soaked for 15 min in the prepared temoporfin solution (10 μg/mL) containing PBS. Temoporfin treatments were used in conjunction with a diode laser (TI-818-1, Transverse Industries Co., Ltd., Taipei, Taiwan). The irradiation distance was 15 cm, and the exposure times were 15 and 30 min (5–10 J/cm^2^) in order to obtain temoporfin-impregnated HM-T15 and HM-T30 hydrogels. The specimen irradiation for 15 (HM-T15) and 30 (HM-T30) min was the same in the physicochemical and biological analyses. However, irradiation was performed after bacterial or cell inoculation and the specimens were then incubated in the dark for 3 h for the biological analysis. All the experiments were handled in the dark.

### 2.3. Solution Absorption of Hydrogels

The HM was immersed in PBS or 10 μg/mL temoporfin. The dried HM samples were weighed (*W*_0_), and the weights (*W_t_*) after immersion for 5, 10, 15, 30, and 60 min were measured. Changes in the weight of the hydrogel were calculated as follows:

(1) Weight varying ratio %=Wt−W0W0 gg×100%

### 2.4. Contact Angle Test

The hydrophilicity of the testing samples (*n* = 5) was determined using a contact angle analyzer (FTA-125, First Ten Angstroms, Portsmouth, VA, USA). The HM was immersed in PBS or 10 μg/mL temoporfin for 15 min, then air dry for 24 h before the contact angle test was performed. A droplet of distilled water was dripped vertically from a 31G needle onto the flat of the specimen at room temperature, and images were recorded by a charge-coupled device (CCD). The contact angle of each water drop was measured using a non-spherical fitting approach.

### 2.5. Fourier Transform Infrared (FTIR) Spectroscopy

Attenuated total reflectance FTIR spectroscopy (Nicolet Is5, Thermo Fisher Scientific, Waltham, MA, USA) was used in order to verify the chemical structure of the hydrogel after cross-linking and to analyze the functional groups of the raw materials.

### 2.6. Fixation Indices of the Cross-Linking Specimens

Ninhydrin (2,2-dihydroxy-1,3-indenedione) (Sigma-Aldrich^®^, St. Louis, MO, USA) was used for amino acid detection. In this study, ninhydrin reacted with amines and amino acids in a colorimetric analysis, which was used to compare the semiquantitative values of the residual functional groups of the active amino acids exposed outside the backbone of hyaluronic acid and gelatin before (*activated amino*)*_fresh_* and after cross-linking through EDC (*activated amino*)*_fixed_*. The content of the unreacted amino acids in the samples was determined using a VersaMax™ ELISA microplate reader (Molecular Devices, San Jose, CA, USA) at 570 nm. One milligram of each hydrogel with and without EDC cross-linking was immersed in deionized water for 1 h. Then, 1 mL of the extract and 0.5 mL of ninhydrin were mixed well, and the mixture was heated to 100 °C for 10 min in order to measure the 570 nm. The fixed index proportional to the degree of cross-linking was calculated [30]:

(2) $$\mathrm{Fixation}\;\mathrm{index}\;\left(\%\right)=\frac{{\left(activated\;amino\right)}_{fresh}-{\left(activated\;amino\right)}_{fixed}}{{\left(activated\;amino\right)}_{fresh}}\times 100\%$$

### 2.7. Degradation Rate of Hydrogels

The sample was pre-immersed in PBS for 24 h, weighed (*W_sat_*), and removed every other day. The excess water on the surface of the specimen was slightly wiped off, and the weight (*W_residual_*) of each specimen was measured until the specimen was fully degraded. Changes in the weights of the specimens were calculated as follows:

(3) $$\mathrm{Rate}\;\mathrm{of}\;\mathrm{weight}\;\mathrm{gain}\;\mathrm{or}\;\mathrm{loss}\;\mathrm{to}\;\mathrm{degradation}\;\left(\%\right)=\left[1-\frac{W_{sat}-W_{residual}}{W_{sat}}\right]\times 100\%$$

### 2.8. Evaluation of the HM with Antibacterial Properties

*Staphylococcus aureus* (ATCC 25923) was cultured in tryptic soy broth (TSB), and 200 μL of the *S. aureus* suspension (2 × 10^4^ CFU/mL) was seeded in a 96-well plate with the hydrogel samples, which were then incubated in the dark at 37 °C for 3 h and exposed for 15 (HM-T15) and 30 min (HM-T30). The kinetic growth curve of *S. aureus* was observed during dark incubation at 37 °C for 24 h. The kinetic analysis included a 5 s shaking step before each of the OD600 time point measurements, which were recorded at 30 min intervals. The concentration was analyzed using a Varioskan LUX multimode microplate reader (Thermo Fisher Scientific Inc., Waltham, MA, USA) [31,32]. 

Bacterial survival in HM, HM-T15, and HM-T30 samples was also assessed via a bacterial viability test. The positive control was ampicillin (0.1 mg/mL). Liquid broth (1.5%) was sterilized in an autoclave, balanced in a water bath at 50 °C for 30 min, and poured into a 10 cm Petri dish in order to obtain solidified TSB agar. Then, 200 μL of the bacterial suspension (2 × 10^4^ CFU/mL) was seeded in a 96-well plate with the hydrogel samples, which were then incubated at 37 °C for 3 h and exposed were 15 and 30 min. Afterwards, 10 μL of the bacterial solution was aspirated, spread on a TSB agar plate, and incubated at 37 °C for 24 h. The visible bacterial colonies on the TSB agar plates were counted. 

### 2.9. Biofilm Formation Assay

One mL of the *S. aureus* suspension (1 × 10^6^ CFU/mL) was inoculated and placed in direct contact with the hydrogel samples in a 24-well plate. After dark incubation at 37 °C for 3 h, the HM-T15 and HMT-30 groups were irradiated for 15 and 30 min, respectively. The positive control was added to ampicillin (0.1 mg/mL). Then, the test samples were incubated in the dark at 37 °C for 24 h. After removing the planktonic bacteria, the biofilms were washed once with sterile phosphate-buffered saline (PBS), and the wells were washed with PBS twice and air-dried for 1 h. Crystal violet (1 mL of 0.1% *w*/*v*) was added to each well, and the plate was kept at 25 °C for 15 min. Then, the crystal violet solution was sucked off, and each well was rinsed with 2 mL of water three to four times. After the water was aspirated, 1 mL of 30% acetic acid was added to each well. The absorbance was determined at 550 nm by using 30% acetic acid in water as a blank on a Varioskan LUX multimode microplate reader [33].

### 2.10. Viability of L-929 Fibroblasts Cultured in Sample Extracts

Mouse fibroblast L-929 was selected and examined in accordance with the cytocompatibility of the ISO 10993-5 guidelines. The L-929 cells were purchased from the Bioresource Collection and Research Center, Taiwan (BCRC number: RM60091). All cell culture media were purchased from Gibco Thermo Fisher Scientific, Inc. (Waltham, MA, USA). The L-929 cells were cultured in Dulbecco’s modified Eagle’s medium (DMEM) containing 10% fetal bovine serum and 1% antibiotic/antimycotic.

The medium group was designated as the control group. The positive controls and the negative controls were 15% DMSO and high-density polyethylene (HDPE) extract, respectively. The experimental groups were administered with hydrogel membrane extracts. The extraction ratio of the HM to the cell culture medium was set to 0.1 g/mL at 37 °C for 24 h. First, 1 × 10^4^ cells/well were inoculated in a 96-well culture plate and incubated for 24 h. Then, the medium was removed, and the sample extract was added to the culture plate. After dark incubation at 37 °C for 3 h, HM-T15 and HMT-30 groups were irradiated for 15 and 30 min, respectively. Then, the culture plates were incubated in the dark at 37 °C for another 24 h, the supernatant was removed, and 100 μL of 3-(4,5-Dimethylthiazol-2-yl)-2,5-diphenyltetrazolium bromide (MTT) reagent (1 mg/mL; M6494, Thermo Fisher Scientific Inc., Waltham, MA, USA) was added to each well, followed by dark incubation at 37 °C for 4 h. The supernatant was removed and washed with 100 μL PBS twice. After the PBS was removed, the blue formazan product was solubilized with 50 μL of DMSO (Mediatech Inc., Manassas, VA, USA) for 15 min. The cell viability was determined using a VersaMax™ ELISA microplate reader at 590 nm.

### 2.11. Proliferation and Attachment of L-929 and D1 on Hydrogels

The L-929 and D1 cell lines were used for the cell proliferation assay and for attachment observation. The D1 cell line was purchased from the American-type Culture Collection, USA (ATCC number: CRL-12424). The D1 cells were cultured in DMEM containing 10% standard fetal bovine serum. The cells (1 × 10^5^ cells/cm^2^) were seeded with hydrogel samples and incubated in the dark at 37 °C for 3 h in 48-well plates. They were then exposed for 15 (HM-T15) and 30 min (HM-T30) and incubated in the dark at 37 °C for 1, 4, 7, 10, and 14 days. At different dark incubation times, cell proliferation was detected using an alamarBlue proliferation assay kit (Bio-Rad AbD Serotec, Berkeley, CA, USA), which was measured at OD570 and OD600 nm using a Varioskan LUX multimode microplate reader.

The cells were washed with PBS for 1 day after 1-day culture and then dehydrated with 2.5% glutaraldehyde, paraformaldehyde, and different alcohol concentrations in order to compare cell morphology and attachment. Afterwards, the hydrogel with cells was gold-plated, and cell morphologies and attachments were observed using a thermal field emission scanning electron microscope (Thermal FE-SEM; JEOL JSM-7800F Prime, JEOL Ltd., Tokyo, Japan). The cell morphology was observed at 500 and 5000× magnification in the same random field.

### 2.12. Pro-Inflammatory Cytokine Gene Expression

The effects of pro-inflammatory-associated gene expression induced by hydrogel in the L-929 cells were observed using reverse transcription-quantitative polymerase chain reaction (RT-QPCR) analyses. First, 1 × 10^5^ cells/cm^2^ were seeded with hydrogel samples and incubated in the dark at 37 °C for 3 h in 48-well plates. They were then exposed for 15 (HM-T15) and 30 min (HM-T30) and were incubated in the dark at 37 °C for 72 h. Then, they were harvested for RT-QPCR analysis. The total RNA from the L-929 was extracted using a TRI reagent (Molecular Research Center, Inc., Cincinnati, OH, USA). The total RNA was reverse transcribed using a random primer, and cDNA was used as a PCR template. The expression of *nitric oxide synthase* (*iNOS*) and pro-inflammatory cytokines, such as *interleukin-1β (IL-1β)* and tumor necrosis factor-α (*TNF-α*), was normalized to *glyceraldehyde 3-phosphate dehydrogenase* (*GAPDH*) expression. The primer sequences of each gene are listed in Table 1. All the tests were conducted in triplicates. Data were analyzed in accordance with previously described methods [33,34].

### 2.13. Statistical Analysis

Differences in the degree of cross-linking, biofilm formation ability, cell viability, cell proliferation, and pro-inflammatory cytokine gene expression were analyzed using an unpaired t-test and a one-way ANOVA in Prism 5.0 (GraphPad Software, Inc., La Jolla, CA, USA). Differences between each variant were considered significant at *p* < 0.05.

## 3. Results

### 3.1. Hydrophobic Temoporfin Did Not Reduce the Water Absorption and Hydrophilicity of HMs

**Figure 1:**
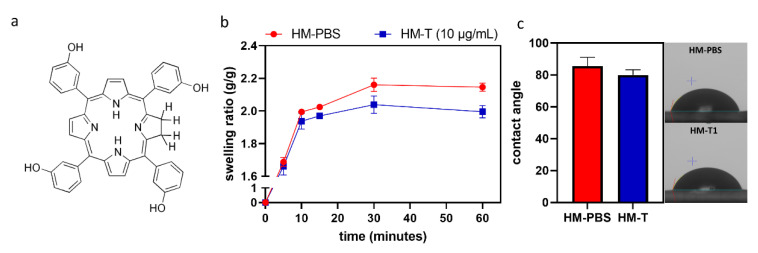
(**a**) Chemical structure of temoporfin (T). (**b**) Measurements of the swelling ratio of hydrogel membranes in terms of their weight rate for 1 h of immersion (*n* = 3). (**c**) Contact angle, *n* = 5. (**b**,**c**) Mann–Whitney test.

Temoporfin is a hydrophobic molecule. Its chemical structure is shown in Figure 1a. The changes in the weight of the HMs within 1 h of immersion in PBS with and without temoporfin (10 μg/mL) are shown in Figure 1b. All the HMs nearly reached water absorption saturation at 30 min and then plateaued. There was no significant difference in the swelling ratio and contact angle (Figure 1c) between the HM-PBS group and the HM-T group. 

### 3.2. Temoporfin and Irradiation Did Not Affect the Cross-Linking Reaction of HM

**Figure 2:**
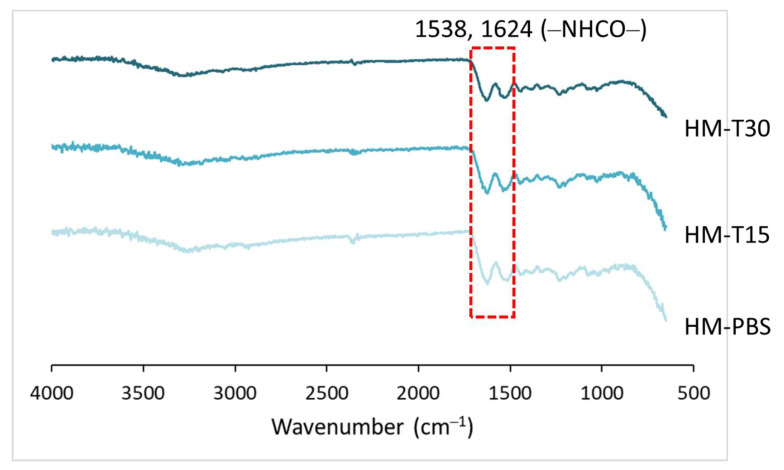
FTIR spectra of hyaluronic acid and gelatin cross-linked with EDC and loaded with temoporfin.

The cross-linking reaction of HM and HM-T specimens were examined using FTIR. The FTIR spectra for the HMs are shown in Figure 2. The amide bond formed via the cross-linking reaction of carboxyl and amine groups by cross-linker EDC had characteristic absorptions at 1538 and 1624 cm^−1^, indicating that the hydrogels were cross-linked [29,35]. The cross-linking was not affected by temoporfin adsorption and red-light irradiation.

### 3.3. Temoporfin and Irradiation Did Not Affect the Fixation Index and Degradation of HM 

**Figure 3:**
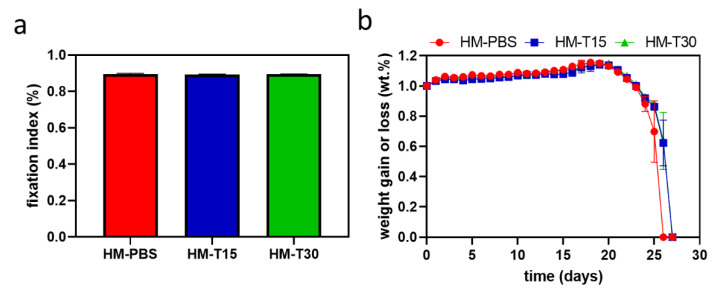
Fixation index (%) reflecting the cross-linking degree of HM, HM-T15, and HM-T30 (*n* = 9, *p* > 0.05). (**a**) Relative long-term immersion demonstrated variation in the weight gain or loss of PBS among the samples (*n* = 6). (**b**) One-way ANOVA.

The amine fixation indices are presented in Figure 3a. The results show that the HM impregnated with temoporfin had a fixation index of >89%. No significant differences were observed between the groups (*p* > 0.05). Therefore, temoporfin used in conjunction with a diode laser did not significantly affect EDC-induced cross-linking. 

A comparison of the changes in the weight of the hydrogel until complete degradation is shown in Figure 3b. The weight change rates for the HMs showed similar trends among the groups. The HMs continued to absorb water slowly after they were soaked for 1 day. The weight of the hydrogels decreased significantly after 20 days of soaking, and degraded completely on day 26. HM-T15 and HM-T30 degraded completely on day 27. Although the hydrophobicity of temoporfin did not affect the surface hydrophobicity of the HM, it caused the inside layer of the HM to be hydrophobic, decreased the swelling rate, and elongated the degradation time of HM-T15 and HM-T30.

### 3.4. Antibacterial Properties of HM with Temoporfin

**Figure 4:**
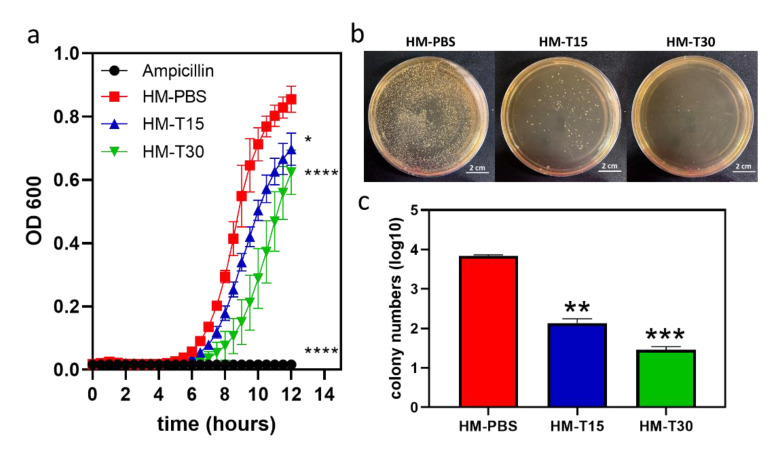
*S. aureus* growth curves for 12 h were analyzed using the kinetic microplate method (*n* = 3). (**a**) Bacterial colonies of different samples cultivated on agar plates for 24 h (*n* = 3). (**b**) Colony numbers. (**c**) One-way ANOVA, * *p <* 0.05, ** *p <* 0.01, *** *p <* 0.001, and **** *p*<0.0001 compared with the HM group.

The 24 h bacterial growth curve of HM and HM-T specimens were examined using the kinetic microplate method. The results showed that the HM had no antibacterial effects against *S. aureus*. The inhibitory activity of temoporfin indicated that increasing the sample release time of HM-T15 and HM-T30 resulted in more evident antibacterial activity at 12 h with varying magnitudes (Figure 4a). However, the final bacterial growth curves of HM-PBS, HM-T15, and HM-T30 all reached the saturation phase at 24 h.

The results of the bacterial viability test (Figure 4b) show that there were fewer colonies of viable *S. aureus* cells in HM-T15 and HM-T30 than in the HM samples. Both HM-T15 and HM-T30 exhibited remarkable antibacterial properties. Figure 4c shows the quantitative results of the colony numbers.

### 3.5. Temoporfin Reduced the Biofilm Formation Ability of S. aureus in HMs

**Figure 5:**
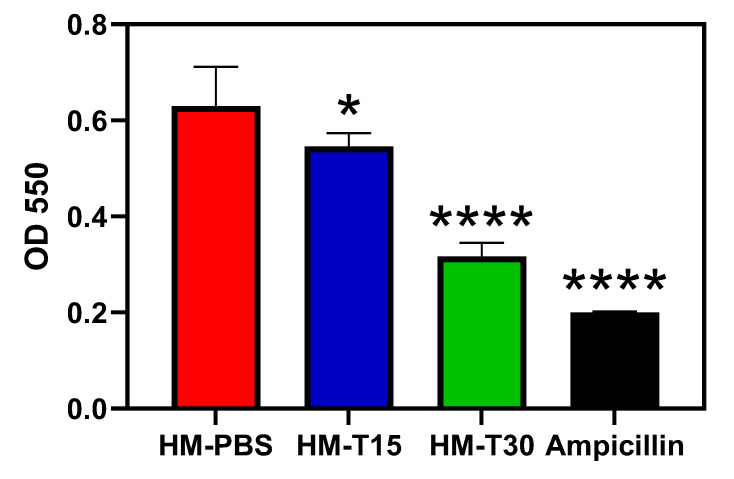
Biofilm-forming ability of *S. aureus* (*n* = 6,). One-way ANOVA, * *p <* 0.05, and **** *p *< 0.0001 compared with the HM group.

The biofilm-forming ability of *S. aureus* on HMs is illustrated in Figure 5. Biofilm formation in the HM-T15, HMT30, and ampicillin groups was significantly lower than in the HM-PBS group (*p* < 0.05). Both HM-T15 and HM-T30 significantly inhibited *S. aureus* biofilm formation.

### 3.6. Cytotoxicity of the HM and HM-T

**Figure 6:**
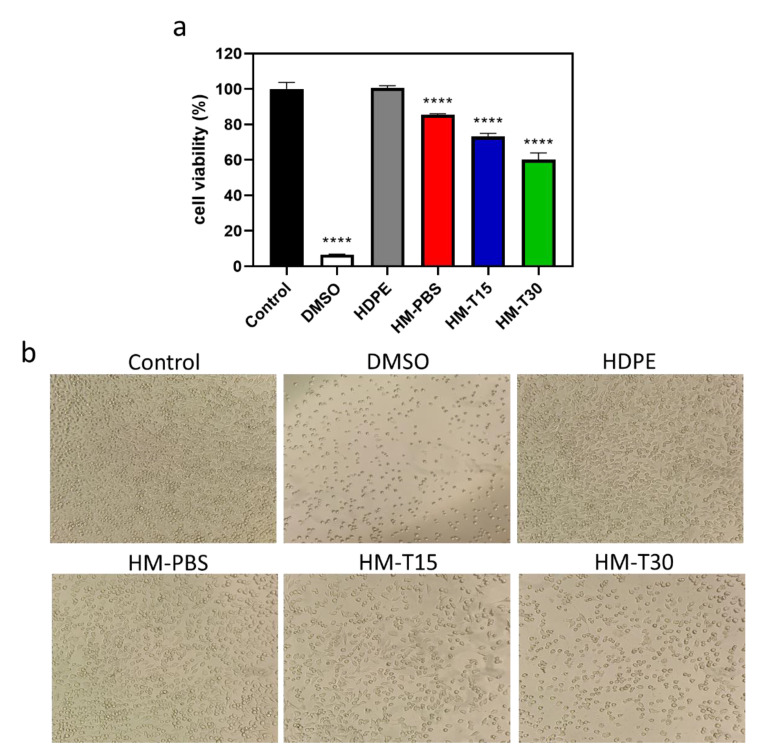
(**a**) Cell viability in the extracted medium of HM, HM-T15, and HM-T30 through 24 h extraction and 24 h L-929 cell culture (*n* = 3). One-way ANOVA, **** *p* < 0.0001 compared with the control group. (**b**) Images of L-929 cells cultured in HM, and HM-T extract for 3 h, and irradiation for 15, and 30 minutes. The images were photographed after 24 h incubation.

The cytotoxicity of the HM and HM-T specimens were examined using the MTT test. The cell viability of the HM (85.41% ± 0.647%) and HM-T15 (73.27% ± 1.286%) were >70%, indicating that a temoporfin exposure time of 15 min affected cell viability and caused cytotoxicity (Figure 6a). The 15 min irradiation time-induced PDT cytotoxicity (HM-T15) still meets the biocompatibility requirements of ISO 10993-5. The cell viability of HM-T30 was only 60.28% ± 3.719% of that of the control group, suggesting that a temoporfin exposure time of 30 min was toxic to the L-929 cells. However, the trend from day 1 to day 14 shows that the cells were still recovering. Elongation of the exposure time was needed in order to control the cytotoxicity and enhance antibacterial activity of temoporfin and to balance and reach the best effects for aPDT therapy. The cells in the control group were healthy and spindle shaped (Figure 6b) after 24 h of dark incubation. In the HDPE exact negative control group, the cells were healthy and spindle shaped, which was similar to those in the control group. This result indicates that sterilization was effective, and no adverse effects on the experimental design were found. The cells in the DMSO positive control group aggregated and were spherical, implying that they lost their metabolic potential. After the cells were incubated in the different groups of extracts for 24 h, those in the HM and HM-T15 groups were spindle shaped, whereas those in the HM-T30 group were spherical. Therefore, temoporfin exposure for 30 min induced cytotoxic effects on the L-929 cells. 

### 3.7. Proliferation of the Hydrogels in Contact with Cells

**Figure 7:**
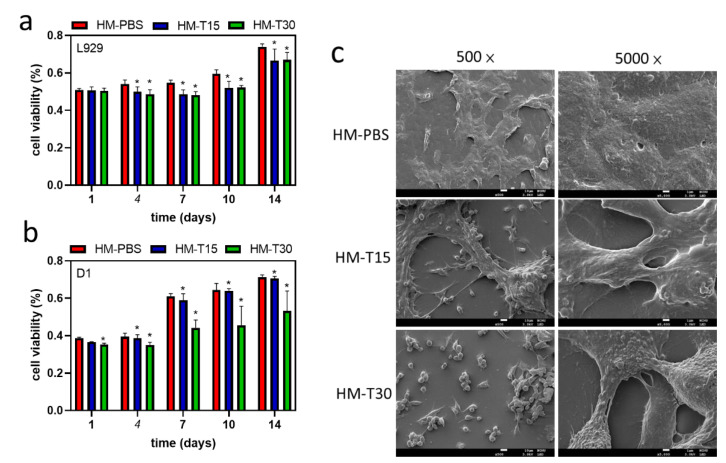
Proliferation of the hydrogel-regenerated membrane in contact with L-929 (**a**) and D1 (**b**) cells for 1, 4, 7, 10, and 14 days. One-way ANOVA, * *p <* 0.05 compared with the HM group at each time point. (**c**) Images of cell attachment on the hydrogel membrane in contact with D1 cells for 1 day.

The L-929 and D1 cells were inoculated and directly attached to the surfaces of different HMs. The hydrogels without temoporfin exhibited good viability on the 14th day of culture (Figure 7a). The expression of cell adhesion and proliferation in the HM group was marginally better compared to the other groups. The cell proliferation in all the hydrogels impregnated with temoporfin significantly slowed down. This was possibly because temoporfin treatment elicits an antibacterial effect that inhibits cell proliferation [31,36].

The cell viability of the different HMs attached to D1 cells is illustrated in Figure 7b. After D1 cells were cultured on the surface of the HMs for 14 days, cell viability was found to have increased with culture time in all the groups. The HM and HM-T15 groups showed better cell survival after dark incubation for 14 days compared to the HM-T30 group. 

The morphological characteristics of the D1 cells attached to the HMs and cultured for 1 day are shown in Figure 7c. For cell adhesion, the cells in the HM group adhered well to the membrane surfaces. However, the cells cultured on HM-T15 and HM-T30 did not attach well because of the inhibitory effects of PDT on the adherent cells. The spherical morphology of the cells was exposure-time-dependent (Figure 7c, 500×). The morphologies of the D1 cells cultured on HM-T15 and HM-T30 were slenderer than those cultured on HM in 24 h of dark incubation (Figure 7c, 5000×). The area of lamellipodia and filopodia decreased. The results of this study revealed that D1 cells were well attached on HMs, but detached on HM-T15 and HM-T30 because the D1 cells were more sensitive to the temoporfin PDT effect than the L-929 cells. Even so, the D1 cells on the surface of HM-T15 and HM-T30 demonstrated continuous growth from day 7 to day 14.

### 3.8. Pro-Inflammatory Cytokine Gene Expression

iNOS, IL-1β, and TNF-α are associated with pro-inflammatory reactions. These gene expression levels were examined using reverse transcription-quantitative polymerase chain reaction (RT-QPCR) in L-929 cells after 72 h of incubation. The mRNA expression of *iNOS* (*p* < 0.05), *IL-1β* (*p* < 0.05), and *TNF-α* (*p* < 0.05) in the HM-T15 and HM-T30 groups was significantly lower than in the HM group. The results indicate that hydrogel containing temoporfin significantly reduced pro-inflammatory reactions.

## 4. Discussion

*S.staphylococci* have been isolated from approximately 50% of patients with gingivitis and periodontitis [37]. Sometimes, the systemic *S. aureus* infection is also mediated by the hyphal invasion of mucosal tissue by *Candida albicans* [38]. Therefore, *S. aureus* can spread to distant cervicofacial regions and cause a systemic infection [39]. Manual ultrasonic instruments, full- and partial-mouth scaling and root planning (SRP), lasers, photodynamic therapy, antibiotics, probiotics, and GTR techniques demonstrate the practical application of current knowledge with respect to the treatment of periodontitis [40,41]. GTR treatment has been used for periodontal defects, including intrabony and localized gingival recession defects. Among the most common treatment methods for chronic periodontitis are effective plaque control methods used to eliminate bacterial infection [42]. After a GTR operation, a systemic antibiotic is usually prescribed to reduce bacterial growth and prevent wound infection [41]. The first step in treating periodontal disease is eliminating bacterial infections. The innate regenerative potential of the periodontium is dependent on wound stability, so the early phase of wound healing is a key point for periodontal tissue repair and regeneration [43]. 

In the present study, hyaluronic acid and gelatin comprised the main structures of the hydrogel membrane, and temoporfin was used to increase the antibacterial function of the hydrogel membrane. We used the principles of aPDT in order to reduce the risk of infection during GTR. The impregnated temoporfin extends the structural stability of the hydrogel (Figure 3b), possibly due to the protective effects of hydrophobic temoporfin under aqueous conditions. This phenomenon may be caused by temoporfin hydrophobicity [17,44]. The temoporfin molecules in the HMs will attach to bacterial membranes. After irradiation, the photosensitizer produced single oxygen molecules and ROS, causing damage to the microbial cell wall [45]. Previously, we found that temoporfin irradiation could be conducted multiple times to inhibit bacterial growth. The minimum inhibitory concentrations (MICs) and minimum bactericidal concentrations (MBCs) of temoporfin were both 2 μg/mL in *S. aureus* under hypoxic conditions [15]. The HM-T15 and HM-T30 groups with temoporfin exerted significant antibacterial effects on *S. aureus* (Figure 4) and likely reduced the risk of bacterial infection in the regenerating areas. Studies have shown that HMs with 10 μg/mL of temoporfin are able to achieve good antibacterial activity. The temoporfin-containing hydrogel membrane in the tissue can repeatedly be given light in order to have multiple bactericidal and bacteriostatic effects. It is important to ensure that local bacterial growth and biofilm formation are inhibited when using the regenerated membrane in order to reduce the use of systemic antibiotics and the risk of systemic infection [15].

After 24 h of cell culture in the extracted HM, HM-T15, and MH-T30 media (Figure 6a), the hydrogel group was treated with temoporfin, which exerted no cytotoxicity when the L-929 cells were exposed for 15 min. For the proliferation of the L-929 and D1 cells attached to HMs for 14 days in the culture, the cell viability increased with culture time in all groups (Figure 7). The trend of L-929 cell viability in the HM-T15 group was different from that of the D1 cells, and the proliferation capability of the L-929 cells on the cell membrane was weaker than that of the D1 cells after day 7. The D1 cells were all in good condition and were firmly attached to the surface of the HM-PBS group (Figure 7c). This result indicates that osteoprogenitor cells (the D1 cell line) attach well and have a greater ability to proliferate in the regenerated membrane with added temoporfin than fibroblast cells (L-929). Before the HM with 10 μg/mL of temoporfin is absorbed, the HM structure may prevent fibroblasts from occupying the growth space of bone cells, which may guide bone regeneration in vivo. 

**Figure 8:**
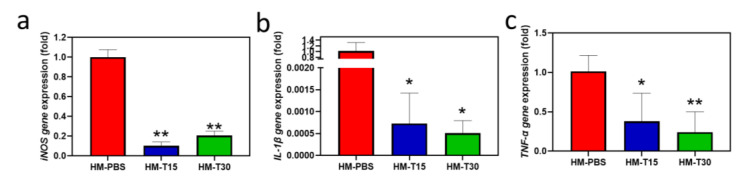
Gene expression of pro-inflammatory cytokines (**a**) *iNOS*, (**b**) *IL-1β*, and (**c**) *TNF-α* in L-929 cells after 72 h. One-way ANOVA, * *p <* 0.05, and ** *p <* 0.01 compared with the HM group.

Persistent inflammation affects regeneration. Consequently, inflammatory responses must be controlled before attempting to aid in regeneration [43,46]. The present study evaluated the effects of *iNOS* and pro-inflammatory cytokines (*IL-1β* and *TNF-α*) with temoporfin hydrogel impregnation on L-929 cells. The HM-T15 and HM-30 groups containing temoporfin significantly reduced pro-inflammatory gene expression (Figure 8), indicating that it could relieve inflammation. Current research is limited to in vitro assays. Even though the immersion of temoporfin in hydrogels delays cell proliferation, it can achieve good antibacterial activity in the early stages of tissue regeneration. This study showed that D1 cells proliferated well only after the culture process was delayed for up to 7 days. Animal models will be used to verify the effects of HMs with 10 μg/mL of temoporfin in further pre-clinical studies.

## 5. Conclusions

In this study, gelatin with hyaluronic acid was used to prepare a hydrogel regeneration membrane that was cross-linked with EDC and further loaded with temoporfin. HM-T15 and HM-T30 with temoporfin exerted significant antibacterial effects against S. aureus. HM-PBS and HM-T15 meet the ISO 10993-5 cytotoxicity requirements. Temoporfin exposure for 15 min did not affect cell proliferation for a long time, and osteoprogenitor cell growth was better than fibroblast cell growth. The HM-T15 and HM-30 groups containing temoporfin exhibited significantly reduced pro-inflammatory gene expression. These membranes had good biocompatibility and inhibited bacterial growth. HM-T15 shows potential as a biodegradable material for biocompatible tissue-guarded regeneration membranes with antibacterial properties. 

## Figures and Tables

**Table 1:** Primer sequences used for pro-inflammatory gene expression.

Gene	Primer Sequence
*GAPDH*	Forward primer: TGGTATCGTGGAAGGACTCATGA Reverse primer: ATGCCAGTGAGCTTCCCGTTCAG
*iNOS*	Forward primer: CCCTTCCGAAGTTTCTGGCAGCAGC Reverse primer: GGCTGTCAGAGCCTCGTGGCTTTGG
*IL-1β*	Forward primer: CCACAGACCTTCCAGGAGAATG Reverse primer: GTGCAGTTCAGTGATCGTACAGG
*TNF-α*	Forward primer: CTCTTCTGCCTGCTGCACTTTG Reverse primer: ATGGGCTACAGGCTTGTCACTC

*GAPDH*: glyceraldehyde 3-phosphate dehydrogenase; iNOS: inducible nitric oxide synthase; *IL*: interleukin; *TNF-α*: tumor necrosis factor-α.

## Data Availability

Not applicable.
